# Targeting natural antioxidant polyphenols to protect neuroinflammation and neurodegenerative diseases: a comprehensive review

**DOI:** 10.3389/fphar.2025.1492517

**Published:** 2025-01-24

**Authors:** Maroua Jalouli, Md Ataur Rahman, Partha Biswas, Hasanur Rahman, Abdel Halim Harrath, In-Seon Lee, Sojin Kang, Jinwon Choi, Moon Nyeo Park, Bonglee Kim

**Affiliations:** ^1^ Department of Biology, College of Science, Imam Mohammad Ibn Saud Islamic University (IMSIU), Riyadh, Saudi Arabia; ^2^ Department of Oncology, Karmanos Cancer Institute, Wayne State University, Detroit, MI, United States; ^3^ Laboratory of Pharmaceutical Biotechnology and Bioinformatics, Department of Genetic Engineering and Biotechnology, Jashore University of Science and Technology, Jashore, Bangladesh; ^4^ Department of Biotechnology and Genetic Engineering, Bangabandhu Sheikh Mujibur Rahman Science and Technology University, Gopalganj, Bangladesh; ^5^ Zoology Department, College of Science, King Saud University, Riyadh, Saudi Arabia; ^6^ College of Korean Medicine, Kyung Hee University, Seoul, Republic of Korea; ^7^ Acupuncture and Meridian Science Research Center, Kyung Hee University, Seoul, Republic of Korea; ^8^ Department of Pathology, College of Korean Medicine, Kyung Hee University, Seoul, Republic of Korea; ^9^ Korean Medicine-Based Drug Repositioning Cancer Research Center, College of Korean Medicine, Kyung Hee University, Seoul, Republic of Korea

**Keywords:** polyphenols, neurodegenerative diseases, neuroinflammatory, signal transduction pathways, therapeutic applications

## Abstract

Polyphenols, naturally occurring phytonutrients found in plant-based foods, have attracted significant attention for their potential therapeutic effects in neurological diseases and neuroinflammation. These compounds possess diverse neuroprotective capabilities, including antioxidant, anti-inflammatory, and anti-amyloid properties, which contribute to mitigating the progression of neurodegenerative conditions such as Alzheimer’s Disease (AD), Parkinson’s Disease (PD), Dementia, Multiple Sclerosis (MS), Stroke, and Huntington’s Disease (HD). Polyphenols have been extensively studied for their ability to regulate inflammatory responses by modulating the activity of pro-inflammatory genes and influencing signal transduction pathways, thereby reducing neuroinflammation and neuronal death. Additionally, polyphenols have shown promise in modulating various cellular signaling pathways associated with neuronal viability, synaptic plasticity, and cognitive function. Epidemiological and clinical studies highlight the potential of polyphenol-rich diets to decrease the risk and alleviate symptoms of neurodegenerative disorders and neuroinflammation. Furthermore, polyphenols have demonstrated their therapeutic potential through the regulation of key signaling pathways such as Akt, Nrf2, STAT, and MAPK, which play critical roles in neuroprotection and the body’s immune response. This review emphasizes the growing body of evidence supporting the therapeutic potential of polyphenols in combating neurodegeneration and neuroinflammation, as well as enhancing brain health. Despite the substantial evidence and promising hypotheses, further research and clinical investigations are necessary to fully understand the role of polyphenols and establish them as advanced therapeutic targets for age-related neurodegenerative diseases and neuroinflammatory conditions.

## 1 Introduction

Neurodegenerative disorders, including Alzheimer’s disease, Parkinson’s disease, and Huntington’s disease, provide a substantial difficulty for healthcare systems worldwide since they worsen over time and there are no effective remedies available (Giri et al., 2024). These incapacitating ailments are distinguished by the slow degeneration of neurons and neural activity, resulting in cognitive deterioration, motor disabilities, and, finally, a substantial fall in the overall wellbeing of the affected persons (Singh K. et al., 2024). Despite extensive research spanning several decades, the hunt for effective treatments for neurodegenerative disorders continues to be a challenging endeavor.

**FIGURE 1:**
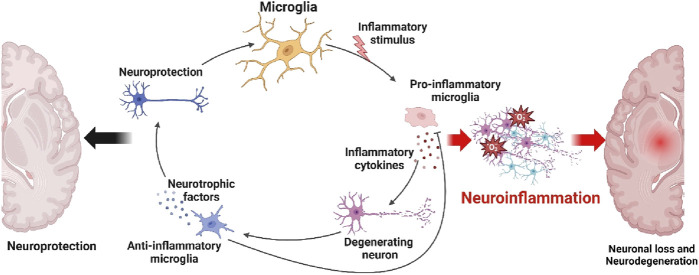
Formation of neuroinflammation and neurodegenerative diseases. Microglia function as both neuroprotective and neurodegenerative agents in the brain’s response to injury and disease. Microglia play a neuroprotective role by promoting neuronal survival and repair by releasing neurotrophic substances and exhibiting an anti-inflammatory phenotype. Microglia, on the other hand, have the ability to become activated in response to pro-inflammatory stimuli like TNF-κ, IL-1β, and IL-6. Neuronal loss and an increasing number of neurodegenerative conditions have been associated with this transformation. Figure drawn by BioRender.com at 26 August 2024.

Neuroinflammation is an essential element in the advancement of neurodegenerative disorders, marked by the stimulation of microglia, the main immune cells comprising the central nervous system (Adamu et al., 2024). Microglia, under normal physiological circumstances, offer neuroprotection by the release of neurotrophic factors and the maintenance of an anti-inflammatory milieu, therefore promoting the wellbeing and optimal functioning of neurons (Maugeri et al., 2021). Nevertheless, when exposed to inflammatory triggers such tumor necrosis factor-alpha (TNF-κ), interleukin-1 beta (IL-1β), and interleukin-6 (IL-6), microglia have the ability to assume a pro-inflammatory configuration (Barry-Carroll and Gomez-Nicola, 2024). These activations trigger the synthesis of cytokines and other inflammatory substances that worsen the damage to neurons, therefore contributing to the death of neurons and the advancement of neurodegenerative disorders (Mesci et al., 2024). Degenerative disorders such as Alzheimer’s, Parkinson’s, and multiple sclerosis are frequently characterized by chronic activation of microglia and persistent inflammation (Thomas et al., 2024). In these conditions, neurodegeneration is driven by a continuous cycle of inflammation and neuronal death. Elucidating the dual function of microglia is crucial for the development of treatments that may alleviate neuroinflammation and enhance neuroprotection in neurodegenerative diseases (Han et al., 2024). Figure 1 depicts the equilibrium between these two microglial immune responses, emphasizing how their level of activation might impact the progression of neuroinflammation and contribute to the development of neurodegenerative disorders.

Polyphenolic compounds, a varied class of naturally occurring bioactive substances, have attracted considerable interest owing to their possible neuroprotective effects (Shi et al., 2024). These chemicals are extensively found in plants, functioning as secondary metabolites that aid in defense systems and coloring. Polyphenols are categorized as flavonoids, phenolic acids, lignans, and stilbenes, each defined by unique structural characteristics (Sejbuk et al., 2024). Flavonoids are categorized as flavones, flavonols, and isoflavones, which are present in fruits, vegetables, tea, and wine (Billowria et al., 2024). Caffeic and ferulic acids, types of phenolic acids, are prevalent in coffee and grains (Kumar et al., 2024). Lignans, found in seeds and whole grains, and stilbenes such as resveratrol, located in grapes and berries, illustrate the structural diversity of polyphenols (Majedi and Ahamad, 2024). This extensive variety of structural kinds highlights their intricate biological functions. Comprehending the origins and structural variety of polyphenolic compounds is crucial for recognizing their antioxidant, anti-inflammatory, and neuroprotective properties, presenting new therapeutic options for neurodegenerative disorders.

There has been an increasing focus on the possible impact of polyphenols in reducing the advancement of neurodegenerative diseases and enhancing brain function (Shi et al., 2024). Polyphenols are inherent substances present in a variety of plant-derived foods, including fruits, vegetables, tea, coffee, and red wine (Thakur and Kumar, 2024). Their antioxidant, anti-inflammatory, and neuroprotective characteristics have attracted attention due to their potential therapeutic benefits in the treatment of neurodegeneration (Shi et al., 2024). Studying plant polyphenols to prevent degeneration and age-related disorders like NDs is part of the search for natural ways to age healthily (Song et al., 2024). *In vitro*, cell-based, animal, and human studies have investigated how dietary polyphenols protect the brain (Figueira et al., 2017).

This research aims to explore the current understanding of the mechanisms through which polyphenols act in neuroinflammation and neurodegenerative diseases, as well as their potential as therapeutic agents. Current preclinical and clinical data that supports the effectiveness of polyphenols in reducing the advancement of diseases, boosting cognitive abilities, and promoting overall brain health (Mayer et al., 2024). In addition, the difficulties and restrictions involved in applying these discoveries to actual medical treatment and suggest future research paths to better understand the healing capabilities of polyphenols in neurodegenerative disorders (Socała et al., 2024). This review aims to provide a clear understanding of how polyphenols and neurodegeneration interact, with the goal of helping to create new treatment approaches to combat these debilitating diseases and enhance the quality of life for millions of affected individuals globally.

## 2 Polyphenols’ pharmacological function to protect neuroinflammation and neurodegenerative diseases

**FIGURE 2:**
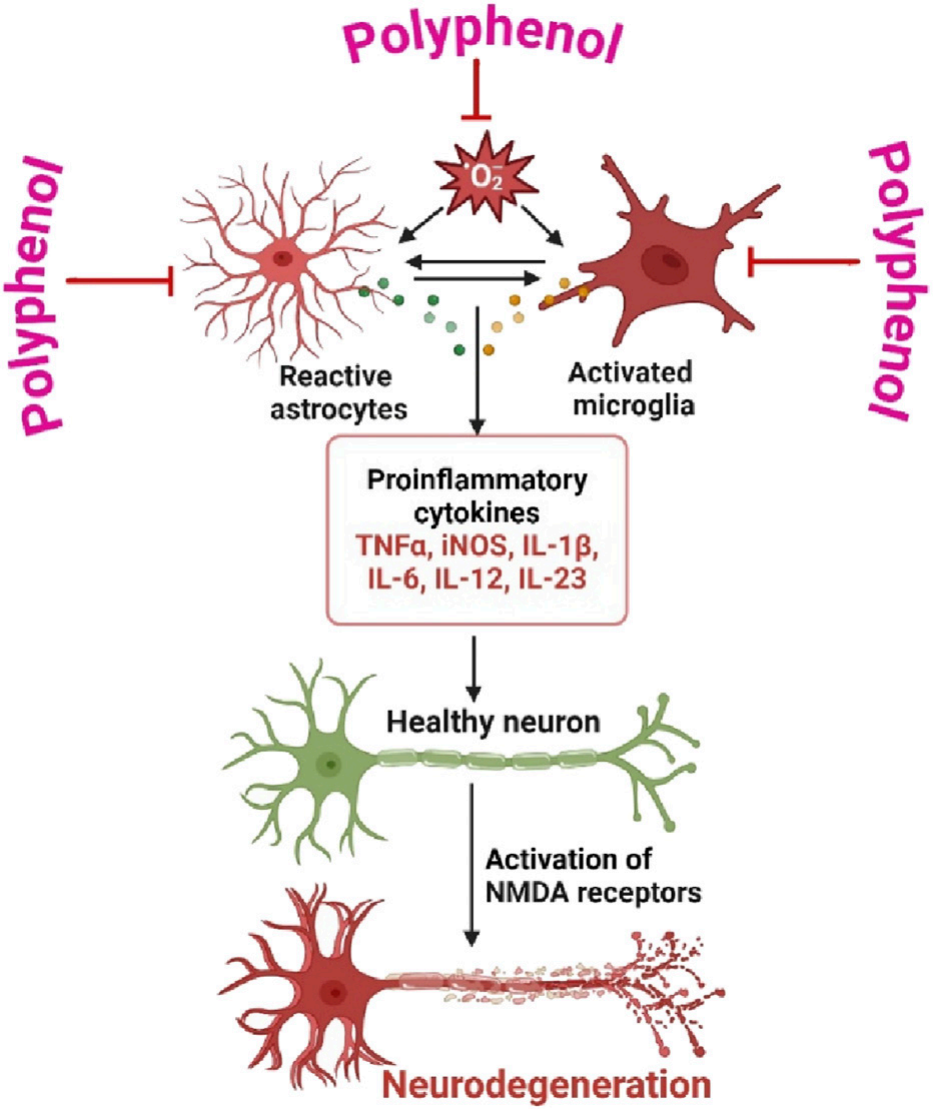
Roles of Astrocytes and Microglia in Neurodegeneration is prevented by polyphenol. The dual functions of astrocytes and microglia in the process of neurodegeneration and the potentially neuroprotective properties of polyphenols. Through the modulation of immunological responses, reduction of oxidative stress, and regulation of inflammatory signaling pathways, polyphenols protect neurodegeneration indicate that polyphenols have the potential to be used as therapeutic agents for neuroinflammation and neurodegenerative diseases. Figure drawn by BioRender.com at 26 August 2024.

Neuroinflammation is a pathological process that involves the activation of glial cells and the subsequent production of pro-inflammatory cytokines, resulting in neuronal damage and impaired functionality (Biswas, 2023). The neuroprotective effects of polyphenols are mediated by many pharmacological mechanisms. One of the principal mechanisms by which polyphenols provide protection against neuroinflammation is the modulation of microglia and astrocytes, which are the immune cells that dwell in the brain (Al Mamun et al., 2024). Polyphenolic compounds, including resveratrol, curcumin, and epigallocatechin gallate (EGCG), have been found to possess inhibitory effects on microglial activation and the production of pro-inflammatory cytokines such as TNF-κ, IL-1β, and IL-6 (Liu et al., 2023). Additionally, these substances facilitate the synthesis of anti-inflammatory cytokines, so fostering a greater equilibrium in the immunological response within the brain (Tayab et al., 2022). Moreover, polyphenols possess robust antioxidant characteristics that greatly contribute to the mitigation of oxidative stress, a state that frequently intensifies neuroinflammation (Figure 2) (Chatterjee et al., 2024). Polyphenols have a crucial role in mitigating oxidative damage to neurons by effectively scavenging reactive oxygen species (ROS) and augmenting the activity of intracellular antioxidant enzymes such as superoxide dismutase (SOD) and glutathione peroxidase (GPx) (Hu et al., 2024). In addition, polyphenols have been observed to engage with signaling pathways implicated in neuroinflammation. One example of its ability is the inhibition of nuclear factor kappa B (NF-κB) activation, which is a crucial transcription factor responsible for regulating the expression of genes associated with inflammation (Vaghari-Tabari et al., 2024). Moreover, polyphenols effectively regulate the mitogen-activated protein kinase (MAPK) pathways, which play a crucial role in cellular reactions to stress and inflammation (Islam et al., 2024). In brief, polyphenols present a potentially effective therapeutic strategy for addressing neuroinflammation through their ability to modulate immunological responses, mitigate oxidative stress, and modulate crucial inflammatory signaling pathways (Dama et al., 2024). Due to their diverse range of effects, they possess the potential to serve as viable candidates in the prevention or deceleration of neurodegenerative disorders. Polyphenols have great potential in preventing and treating neurological conditions because of their diverse pharmacological properties, which include antioxidant, anti-inflammatory, and neuroprotective effects. Their possible neuroprotective properties, especially in the context of neurodegenerative disorders like Alzheimer’s, Parkinson’s, and Huntington’s diseases, have attracted considerable study. Table 1 is a representation of the pharmacological function of polyphenols under different neurodegenerative diseases.

**TABLE 1:** The tabular representation of the pharmacological role of polyphenols in neurodegenerative diseases.

S.N.	Name of the polyphenols	Potential activity	Test system	Test dose	Targeted pathways	Potential mechanism	References
01	Curcumin	Parkinson’s disease (PD)	N27 dopaminergic neuronal cell cultures	0–1,000 μM	Peroxynitrite-mediated nitrosative stress	Protects from inhibition (leading to mitochondrial dysfunction) and NS with mitochondrial complex	Chithra et al. (2023)
02	Curcumin	Neurodegenerative diseases	Neurotoxicity in rats caused by homocysteine	5 and 50 mg/kg	Homocysteine-induced cognitive impairment and oxidative stress	Reduces peroxidation of lipid and Enhances memory and learning in rats	Plascencia-Villa and Perry (2023)
03	Curcumin	Neurodegenerative diseases	Neurotoxicity 3-Nitropropionic acid (3-NP) caused in rats	20 mg/kg	3-nitorpropionic acid-induced neurotoxicity	3-NP-induced OS is reduced (including lipid peroxidation, reduced GSH and nitrite activity)	Banji and Banji (2024)
04	Epigallocatechingallat (EGCG)	Neurodegenerative diseases	Immortalized rat neurons (H 19-7)	10–200 mM	Oxidative Stress	Improves cell resistance to oxidative damage by glucose oxidase	Singh et al. (2024b)
05	Epigallocatechingallat (EGCG)	Parkinson’s disease	Immortalized hippocampal neuronal cell line from mice	0.36 mM	Neurodegeneration and Levodopa Methylation	Reduces oxidative cytotoxicity and inhibits the NF-kB signalling pathway	Kang et al. (2010)
06	Epigallocatechingallat (EGCG)	Cerebral Ischemia	International cerebral ischemia C57BL/6 mice	50 mg/kg	MMP-9 activity is upregulated and neuronal cell damage is minimized	MMP-9 activity is upregulated and neuronal cell damage is minimized	Ye et al. (2024)
07	Epigallocatechingallat (EGCG)	Age-mediate oxidative damage	In the rat brain, age-related oxidative damage	2 mg/kg body weight/day	Senescence-induced oxidative exacerbations	SOD, catalase, glutathione peroxidase, glutathione reductase, and glucose-6-phosphate dehydrogenase all benefit from this supplement	Mokra et al. (2022)
08	Mangiferin	Neurodegenerative disorders	Neurotoxicity of glutamate-induced cortical neurons in rats model	2.5 mg/mL	Glutamate-induced neurotoxicity	Inhibit neuronal damage, oxidative stress and depolarization	Walia et al. (2021)
09	Mangiferin	Parkinson’s disease	Murine neuroblastoma cell line N2A	0.25 mM	Toxicity of oxidative stress-mediated 1-methyl-4-phenylpyridinium	Recovers GSH material and decreases the expression of SOD as well as mRNA catalase	Amazzal et al. (2007)
10	Mangiferin Morin	Ischemic brain damage	Neurotoxicity in the primary rat culture of neurons caused by glutamate	100 nM	Reactive oxygen species formation and enzyme antioxidant system activation	Reduces ROS development and stimulates the mechanism of enzyme antioxidants	Alberdi et al. (2018)
11	Resveratrol	Alzheimer’s and Parkinson’s disease	Dopaminergic neurodegeneration in rats due to lipopolysaccharide (LPS)	15–60 μM	Lipopolysaccharide-Induced Neurotoxicity	Reduces ROS-mediated NADPH oxidase activity and delays MAPK and NF-kB activation pathways	Teijido and Cacabelos (2018)
12	Resveratrol	Ischemic-reperfusion stroke	Optimized form of ischemical reperfusion in mouse	25 μM	Glutamate-induced toxicity	Protects mouse neurons that are exposed to an optimized ischemical stroke	Fan et al. (2024)
13	Resveratrol	Atherosclerosis	Tetrahydropyridine (MPTP)- Parkinson mediated in mouse	30 mg/kg	MPTP-Induced Neuronal Loss	Protects mouse neurons that are exposed to an optimized ischemical stroke	Liang et al. (2024)
14	Resveratrol	Dopaminergic neuronal damage	Midbrain slice culture dopaminergic neurons	30 and 100 μM	ROS signaling and cellular glutathione	Prevents accumulation of ROS, cellular glutathione, and cellular oxidative damage from MPP(+)	Feng et al. (2024)
15	Resveratrol	Alzheimer’s Disease	Alzheimer’s Mouse Model (Tg2576 line) (AD)	5 µM	Amyloid-β Toxicity	Peroxiredoxins and mitochondrial structural genes are expressed normally	Lymperopoulos et al. (2024)
16	Tea Polyphenol	Brain excitotoxic injury	NMDA-induced neurotoxicity in mice	60 mg/kg/day	Brain Excitotoxicity	Reduces synaptosomal ROS development	Niinuma et al. (2024)

### 2.1 Potential role in Alzheimer’s disease and dementia

In AD and Dementia, polyphenols have been demonstrated as a vital neuroprotective characteristic evolving the therapeutic option. Green tea and White tea extracts have been used as a therapeutic goal in treating neurodegenerative age-medicated diseases like AD and dementia to inhibit acetylcholinesterase activity (Afzal et al., 2022). In the Aβ-mediated cytotoxicity in the rat model, the polyphenols of green tea regulated the expression of initial rat cortical neurons (Albadrani et al., 2024). A research study has demonstrated that the grape’s polyphenols enhanced the cognitive efficacy in the mouse research model developed of AD (Tavan et al., 2024). The synaptic transmission was enhanced by the epicatechin metabolite 3′-O-methyl-epicatechin-5-O-*β*-glucuronide via the response binding protein of cyclic adenosine monophosphate (cAMP) (Jaeger et al., 2018). A research study has revealed that in the transgenic mice research model, the suppression of oligomerization of Aβ peptides has been exhibited by the polymeric polyphenol of grape seed and regulated cognitive losses neuronal reduction cells (Jadidian et al., 2024). The same research study also reported that the grape’s polyphenols showed the potential activity to minimize the abnormality for tau protein folding (Jadidian et al., 2024). Many experiments with animal models have reported that grape seed polyphenols suggested anti-Aβ activity (Al Amin et al., 2024; Vicente-Zurdo et al., 2024).

**FIGURE 3:**
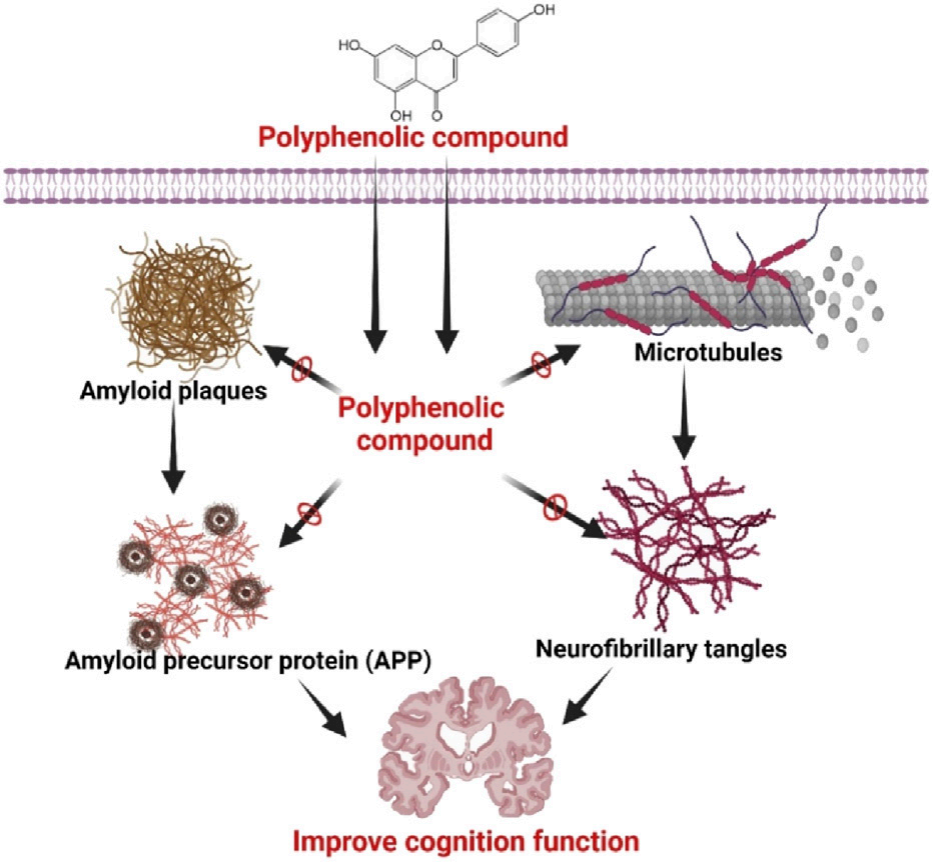
Effects of polyphenolic compounds in AD to improve cognition function. Polyphenolic substances have been found to possess inhibitory effects on the production of amyloid plaques, microtubule stabilization, and modulation of amyloid precursor protein (APP) processing, resulting in a reduction in neurofibrillary tangle deposition. The aforementioned acts collectively lead to the enhancement of cognitive function in individuals with neurodegenerative diseases by which polyphenolic compounds demonstrate their neuroprotective properties, emphasizing their potential as therapeutic interventions in the treatment of cognitive decline linked to conditions like Alzheimer’s disease. Figure drawn by BioRender.com at 26 July 2024.

Resveratrol, an enormous polyphenol in the red wines and grapes, suppressed the expression of Aβ 42 fibril and regulated the Aβ neurotoxicity through suppressing the inhibition of the induceable nitric oxide synthase (Shah et al., 2024). In AD, the Resveratrol exhibited high therapeutic’s activity in the lipid core nanocapsules. It has been observed that the flavonoid fisetin suppressed Aβ fibril expression, and this evidence indicated that the flavonoid fisetin has appeared as a potential therapeutics for the treatment of AD (Al Amin et al., 2024; Bagchi et al., 2020). Morin formed by 2′,3,4′,5,7-pentahydroxyflavone has revealed a potent neuroprotective activity to inhibit neurons’ death by suppressing the tau hyperphosphorylation initiated by the Aβ (Yilmazer et al., 2024). Likewise, a research study on the transgenic mouse model, the tannic acid, has been noted to inhibit Aβ deposition by reducing the fission of β carboxyl-terminal amyloid precursor protein (APP) complex and regulated the inflammation of neuron cells (Manoharan et al., 2024). A research study in the 5XFAD transgenic mouse research model, A flavonoid named 7,8-dihydroxyflavone, has been identified to develop the cognitive efficacy to decrease the secretion of β-secretase enzyme and the synthesis of amyloid-beta (Aβ through the activation of receptor tyrosine kinase B (Emili et al., 2022). Moreover, the polyphenol liquiritigenin developed memory in the Tg2376 mice research model with AD (Zhong et al., 2024). It also minimized the astrocytosis and reduced the activation of Notch-2 for that it can regulate neuron death (Onciul et al., 2024). Figure 3 represents the overall mechanism of polyphenol in AD.

Curcumin protects against peroxynitrite-mediated nitrosative stress and mitochondrial dysfunction in N27 dopaminergic neuronal cell cultures exposed to 0–1,000 μM concentrations, mitigating the inhibition of mitochondrial complexes (Chithra et al., 2023). Curcumin in neurodegenerative diseases: In a study on neurotoxicity in rats induced by homocysteine, curcumin administered at doses of 5 and 50 mg/kg demonstrated protective effects (Plascencia-Villa and Perry, 2023). It alleviated homocysteine-induced cognitive impairment and oxidative stress, reduced lipid peroxidation, and improved memory and learning in rats (Banji and Banji, 2024). Epigallocatechin gallate (EGCG) enhances resistance to oxidative damage induced by glucose oxidase in immortalized rat neurons (H 19-7) at concentrations ranging from 10 to 200 mM, demonstrating its potential in addressing oxidative stress in neurodegenerative diseases (Singh S. et al., 2024). EGCG was tested in an immortalized hippocampal neuronal cell line derived from mice to study its effects on Parkinson’s disease. At a concentration of 0.36 mM, EGCG was found to reduce oxidative cytotoxicity, inhibit the NF-κB signaling pathway, and counteract neurodegeneration and levodopa methylation (Kang et al., 2010). EGCG helps mitigate age-related oxidative damage in the rat brain. Administered at a dose of 2 mg/kg body weight per day, it alleviates senescence-induced oxidative stress and enhances the activity of antioxidants such as superoxide dismutase (SOD), catalase, glutathione peroxidase, glutathione reductase, and glucose-6-phosphate dehydrogenase (Mokra et al., 2022) (Ye et al., 2024). Mangiferin (2.5 mg/mL) has been shown to inhibit neuronal damage, oxidative stress, and depolarization in a rat model of glutamate-induced neurotoxicity in cortical neurons, suggesting its potential therapeutic effects in neurodegenerative disorders (Walia et al., 2021). Mangiferin in a murine neuroblastoma cell line (N2A) at a concentration of 0.25 mM protects against oxidative stress-induced toxicity caused by 1-methyl-4-phenylpyridinium. It restores GSH levels and reduces the expression of superoxide dismutase (SOD) as well as the mRNA levels of catalase (Amazzal et al., 2007). Mangiferin and morin reduce reactive oxygen species (ROS) formation and activate the enzyme antioxidant system in primary rat neuron cultures exposed to glutamate-induced neurotoxicity. At a concentration of 100 nM, they help mitigate ischemic brain damage by stimulating the antioxidant enzyme mechanisms (Alberdi et al., 2018). Resveratrol has been shown to reduce dopaminergic neurodegeneration in rats induced by lipopolysaccharide (LPS) at concentrations ranging from 15 to 60 μM. It mitigates lipopolysaccharide-induced neurotoxicity by decreasing ROS-mediated NADPH oxidase activity and delaying the activation of MAPK and NF-kB pathways (Teijido and Cacabelos, 2018). Resveratrol protects dopaminergic neurons in midbrain slice cultures from damage induced by MPP(+), by preventing the accumulation of reactive oxygen species (ROS) and cellular oxidative damage, through modulation of ROS signaling and maintaining cellular glutathione levels at concentrations of 30 and 100 μM (Feng et al., 2024). Tea polyphenol (60 mg/kg/day) reduces synaptosomal ROS development and mitigates brain excitotoxic injury, including NMDA-induced neurotoxicity in mice (Niinuma et al., 2024).

In AD, it has been noted that the quercetin and rutin decreased the Aβ formation and also inhibited the aggregation of Aβ fibrils (Monteiro et al., 2024). The animal model studies revealed that both compounds also inhibited scopolamine-mediated amnesia. Research with flavonoid rutin has been shown in neuroblastoma cells SH-SY5Y, which can regulate oxidative stress, malondialdehyde and glutathione disulfide (Amiri et al., 2024). It also reduced the activation of inflammatory cascade by reducing cytokines such as TNF-α and IL-1β. Ferulic acid is a phenolic acid that has been revealed as a higher neuroprotective agent to regulate the Aβ toxicity than the polyphenol quercetin (Mugundhan et al., 2024). Many research studies have recently documented that polyphenols provide therapeutic potential in both the cell line and the animal research model. Polyphenols are proposed as a treatment target for age-related diseases such as AD and dementia for their efficiency to develop synaptic transmission by regulating cAMP, Aβ toxicity, and various signaling pathways (Dhapola et al., 2024).

### 2.2 Potential role in Parkinson’s disease (PD)

**FIGURE 4:**
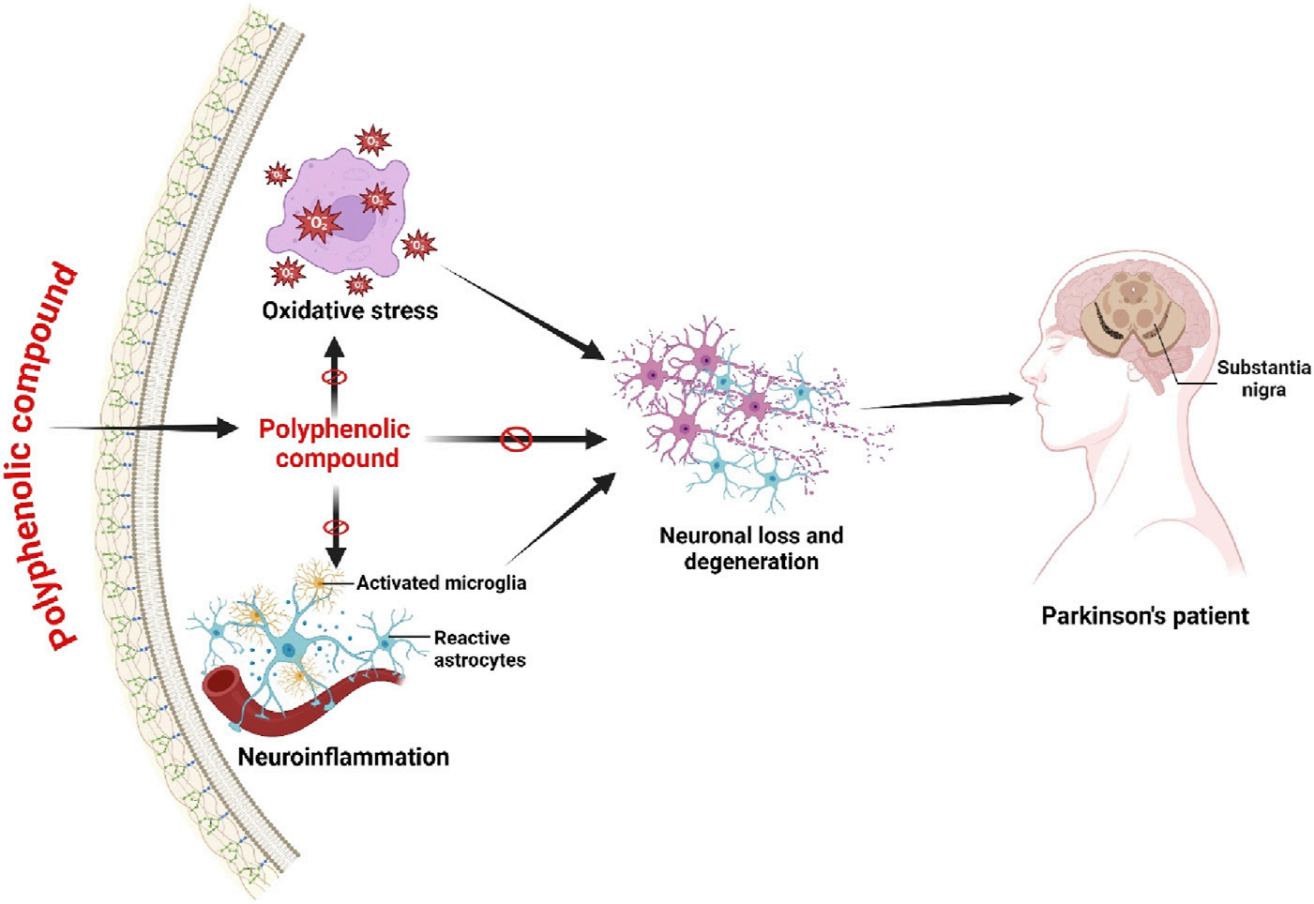
Effects on polyphenolic compound in Parkinson’s Disease. Polyphenolic chemical exerts inhibitory effects on oxidative stress, hence inducing the activation of microglia, thereby mitigating neuronal death. The mechanism by which Parkinson’s Disease progression is inhibited involves the lowering of oxidative stress and the protective function of activated microglia. Figure drawn by BioRender.com at 26 July 2024.

Predominantly, Parkinson’s Disease (PD) is associated with oxidative stress and inflammation that decreases the number of dopaminergic neurons (Watanabe et al., 2024). Polyphenols have been recognized as a therapeutic target for treating PD to reduce inflammation and oxidative stress (Gahtani et al., 2024). Resveratrol can regulate the reduction of dopaminergic neurons in the rat research model with PD (Zhang Y. et al., 2024). The research studies have noted that in PD, through reducing the mRNA expression of cyclooxygenase-2 (COX-2) and the mRNA of TNF-α in substantia nigra, the resveratrol has been exhibited to inhibit the neural inflammation (Xue et al., 2024). Besides, in the rat research model of PD, resveratrol has shown the potential activity to reduce oxidative stress, protein carbonyl (PC), and lipid peroxidation (Khan et al., 2024). Many research studies have shown that the action of oxyresveratrol also reduces neuronal loss in SH-SY5Y cells by reducing SIRT1 and caspase-3, c-jun transcription factors and c-jun N-terminal kinase (JNK) (Rahman et al., 2017; Rahman et al., 2021). Likewise, oxyresveratrol, the ferulic acid, also exhibited the potential neuroprotective activity through down-regulating the JNK pathway (Caban et al., 2023). The reduction of 1-methyl-4-phenylpyrimidinium (MMP) regulated the microglia activation used as a precursor for PD’s pathogenesis, and these abnormalities were overcome by quercetin (Bournival et al., 2012). Studies have demonstrated that quercetin can show the potential neuroprotective activity in mice model with PD through augmenting glutathione peroxidase (G{X), Na (+), K (+) -ATPase and superoxide dismutase (SOD) (Gugliandolo et al., 2020; Biswas et al., 2022). Other studies have shown that quercetin has prevented cell death in the PD mice research model, however, when quercetin is used as its metabolite known as quercetin-3-O-β-glucuronide due to its lower absorption rate (Muñoz-Reyes et al., 2022). Many other research studies in PD have demonstrated that the high neuroprotective activity can be induced by the other polyphenols known as kaempferol, baicalein, EGCG, and caffeic acid (Tavan et al., 2024; Bhullar and Rupasinghe, 2013). Identically, the polyphenols derived from various plant extracts have also shown the neuroprotective pharmacological role in PD research studies (Figure 4).

### 2.3 Potential role of polyphenols in multiple sclerosis (MS)

Multiple sclerosis (MS) is a neurodegenerative disease characterized by autoimmune-related demyelination of the central nervous system (CNS), which can result in cognitive impairment and paralysis. The inflammation and downregulation of immunity can be attenuated by MS therapies (Zeng et al., 2024). In the experimental autoimmune encephalomyelitis (EAE) research model of MS, the inhibition of neuron cell loss without any immunosuppression has been exerted by the polyphenol resveratrol through tracing the 2 homolog1 (SIRT1) activator (Azam et al., 2019). The formulation of therapeutic’s standard of resveratrol SRT501 was shown to reduce the neuron cell damage in the EAE cell line by activating SIRT1 (Chen et al., 2024a). Cell culture-based research studies by resveratrol have revealed SIRT1-associated neuroprotective activity (Yahia et al., 2024; Chen et al., 2013). The polyphenol quercetin showed the immune regulation activity through regulating the expression of TNF-α and IL-1β and also decreased the proliferation rate in a peripheral based mononuclear cell which derived from the various sclerosis patient’s body (Ferreira et al., 2024). The epigallocatechin-3-gallate (EGCG) noted the strong neuroprotective activity via controlling the neuro-inflammation and reducing the damage of neuron cells (Tripathi et al., 2024). Moreover, the other polyphenols like piceatannol, apple polyphenols 222, myricetin, and quercetin have regulated the activation of SIRT1 and shown effective therapeutic potential in MS treatment (Rudrapal et al., 2024). Polyphenols have been suggested as a strong therapeutic target for the treatment of age-related multiple sclerosis (MS) and amyotrophic lateral sclerosis (ALS) due to their potential properties (ALS) (Chatterjee et al., 2024).

## 3 Molecular mechanism of polyphenols based on cell signaling in neuroinflammation and neurodegenerative diseases

Polyphenols, a heterogeneous collection of naturally derived substances present in plants, have attracted interest due to their possible therapeutic properties, specifically in the context of neurodegenerative disorders. The molecular mechanism of polyphenols in neurodegenerative diseases is based on cell signalings are presented in Table 2 is a list of frequently encountered polyphenols, along with their respective plant origins, usual levels of concentration, and their molecular mechanisms involved in neurodegeneration. Their modes of operation frequently entail the regulation of cell signaling pathways that are vital for cellular function and survival. These are the main factors by which polyphenols can affect these pathways.

**TABLE 2:** Polyphenols are a varied collection of naturally existing chemicals present in plants, renowned for their antioxidant characteristics and possible therapeutic impacts on neurodegenerative disorders.

Polyphenol	Plant source	Typical concentrations	Molecular action in neurodegeneration	References
Resveratrol	Grapes, berries	1–50 µM	Antioxidant, anti-inflammatory, enhances autophagy, upregulates SIRT1 and BDNF	Mohammadi et al. (2024)
Curcumin	Turmeric	1–20 µM	Antioxidant, anti-inflammatory, modulates NF-κB and Nrf2 pathways, promotes autophagy	Xu et al. (2024)
Epigallocatechin gallate (EGCG)	Green tea	5–100 µM	Antioxidant, reduces oxidative stress, modulates PI3K/Akt pathway, inhibits Aβ aggregation	Tripathi et al. (2024)
Quercetin	Onions, apples	10–50 µM	Antioxidant, anti-inflammatory, inhibits MAPK pathway, protects against Aβ toxicity	de Oliveira Vian et al. (2024)
Kaempferol	Kale, spinach	1–20 µM	Antioxidant, anti-inflammatory, inhibits pro-inflammatory cytokines, enhances autophagy	Chen et al. (2024b)
Luteolin	Celery, parsley	5–50 µM	Antioxidant, anti-inflammatory, inhibits NF-κB, reduces neuroinflammation	Charrière et al. (2024)
Apigenin	Chamomile, parsley	1–20 µM	Antioxidant, anti-inflammatory, modulates MAPK/ERK pathway, promotes neurogenesis	Aprotosoaie and Miron (2023)
Hesperidin	Citrus fruits	10–100 µM	Antioxidant, anti-inflammatory, modulates Nrf2 pathway, enhances BDNF expression	Kwatra et al. (2020)
Naringenin	Grapefruit, oranges	10–50 µM	Antioxidant, anti-inflammatory, inhibits NF-κB, promotes neuroprotection	Atoki et al. (2024)
Fisetin	Strawberries, apples	5–50 µM	Antioxidant, enhances autophagy, promotes synaptic plasticity, increases BDNF	Ravula et al. (2021)
Genistein	Soybeans	1–50 µM	Antioxidant, modulates estrogen receptors, inhibits tyrosine kinase activity, promotes neuroprotection	Schreihofer and Oppong-Gyebi (2019)
Myricetin	Berries, grapes	10–50 µM	Antioxidant, anti-inflammatory, inhibits Aβ fibrillization, modulates PI3K/Akt pathway	Vauzour (2012)
Catechin	Green tea, cocoa	10–100 µM	Antioxidant, modulates Nrf2 pathway, inhibits Aβ toxicity, enhances synaptic function	Sebastiani et al. (2021)
Baicalein	Scutellaria baicalensis	1–20 µM	Antioxidant, anti-inflammatory, inhibits Aβ aggregation, promotes autophagy	Li et al. (2017)
Rutin	Buckwheat, citrus fruits	10–100 µM	Antioxidant, reduces oxidative stress, inhibits pro-inflammatory cytokines, promotes neuroprotection	Prasad and Prasad (2019)
Pterostilbene	Blueberries	1–50 µM	Antioxidant, anti-inflammatory, modulates SIRT1, enhances cognitive function	Zhu et al. (2022)
Caffeic acid	Coffee, berries	10–100 µM	Antioxidant, anti-inflammatory, inhibits NF-κB, promotes neurogenesis	Colombo and Papetti (2020)
Ellagic acid	Pomegranates, berries	10–50 µM	Antioxidant, inhibits Aβ aggregation, modulates PI3K/Akt pathway, enhances autophagy	de Oliveira (2016)
Silymarin	Milk thistle	10–100 µM	Antioxidant, anti-inflammatory, inhibits NF-κB, promotes neuroprotection	Surai (2015)
Apigenin	Chamomile, parsley	1–20 µM	Antioxidant, anti-inflammatory, modulates MAPK/ERK pathway, promotes neurogenesis	Aprotosoaie and Miron (2023)

### 3.1 Antioxidant activity

Polyphenols possess the ability to eliminate harmful free radicals and enhance the activity of natural antioxidant enzymes such as superoxide dismutase (SOD), catalase, and glutathione peroxidase (Jomova et al., 2024; Dzah et al., 2024). This decreases oxidative stress, which is involved in the development of neurodegenerative disorders like Alzheimer’s and Parkinson’s diseases (Gahtani et al., 2024; Wang J. et al., 2024).

### 3.2 Anti-inflammatory effects

Neurodegenerative diseases are significantly influenced by inflammation. Polyphenols have the ability to hinder pro-inflammatory substances including NF-κB, COX-2, and iNOS, and decrease the generation of cytokines such as TNF-α, IL-1β, and IL-6 (Prakash et al., 2024; Nery‐Flores et al., 2024). This action aids in regulating the neuroinflammatory response, which has the potential to decelerate the progression of the disease.

### 3.3 Modulation of signal transduction pathways

Polyphenols exert an impact on various signaling pathways, encompassing.

#### 3.3.1 MAPK pathway

The MAPK pathway involves the regulation of mitogen-activated protein kinases (MAPKs), which can have an impact on cell survival, differentiation, and apoptosis (Ampadu et al., 2024). Both the nervous system and other tissues benefit from the ERK1/2 and PI3K pathways. The ERK is found in several involving physiological functions such as proliferation, differentiation, and controlling various growth factors’ response (Islam et al., 2024). With the aid of upstream activator kinase and mitogen-activated protein kinase (MEK), phosphorylation of threonine and tyrosine residues activates ERK1/2. ERK1/2 changes its position after activation and phosphorylates a variety of molecules, including transcription regulatory and cytoskeletal protein. Some phenolic compounds activate the ERK1/2, which enhances cell survival in several cell lines. The protection of PC12 cell against apoptosis was observed upon the activation of ERK1/2 by lutein (Hu et al., 2021). Furthermore, the viability of PC12 cells treated with U0126, an ERK1/2 kinase inhibitor, was decreased. ERK1/2 is important to understand the neuronal differentiation and activation of the released cytoskeleton and synaptic protein (Waetzig et al., 2017). Luteolin has been identified to improve outgrowing neuritis and expression of GAP-43 protein, known as neuronal biomarkers, and (HO-1) oxygenase1 expression in PC12 cells (Jain and Jain, 2019). The pharmacological inhibition of ERK 1/2 can block both of these effects. This research study has also shown that both ERE and PKC are involved in the production of PC12 neuritis (Bhuia et al., 2024). Simmilarly, the involvement of ERK and PKC pathways in the neurogenesis phase in PC12 cells also leads to a curcurrination response. The ERK and P38MAPK also inhibit Artepillin C-induced neutric outgrowth of PC12m3 cells (Motoda et al., 2019).

**FIGURE 5:**
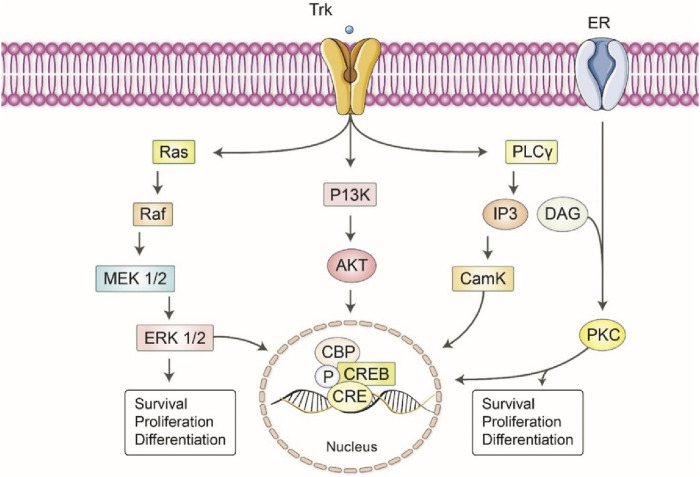
Principal signals that mediate the neurotrophic effects of different polyphenols. The neuromodulatory effects of different polyphenols are facilitated by several intracellular signaling pathways. The activation of the RAS/Raf/MEK pathway by polyphenols results in the modulation of neuronal survival and differentiation. Furthermore, the PI3K/AKT pathway is activated, hence facilitating cellular viability and proliferation. The IP3/PKC signaling cascade is of paramount importance in the mechanism of calcium signaling and the preservation of neuronic integrity. The combined actions of these pathways play a significant role in the neuroprotective effects of polyphenols, underscoring their potential therapeutic applications in the treatment of neurodegenerative disorders.

The activation of P38 MARK by ERK was also shown to be responsible for the c-inducing artepillin neuritis outgrowth of PC12m3 cells (Kano et al., 2008) (Figure 5). Finally, fisetin has been demonstrated that works efficiently during PC12 differentiation, while MEK inhibitors have reduced the fisetin induced ERK and neuritis outgrowth (Anwar et al., 2021).

#### 3.3.2 Activation of PI3K/Akt pathway

Stimulating this route can enhance cell viability and protect against neurodegeneration, which is essential for preventing neuronal death (Manju and Bharadvaja, 2024). Recent studies suggest that PI3K and AKt (PI3K downstream effector) are involved in the survival of neurons (Ma et al., 2024). It also enhances the outgrowth of neurite and other neurotropic effects of polyphenols (Tavan et al., 2024). A research study has demonstrated that PI3K/AKt was responsible in Aβ induced cognitive impairment in mice (Liu G. et al., 2024). By increasing the neurotrophins via PCREB and pAKt signaling, it may be possible to protect neurons against hypoxia (Tregub et al., 2024). Methyl 3, 4-dihydroxybenzoate, a chemical compound derived from phenolic acid, can improve survival and neuronal outgrowth in cultivated cortical neurons via PI3K/AKt signaling pathway (Gopathy et al., 2024). The promotion of neuronal survival and neurite outgrowth may be interrupted by a PI3K specific inhibitor (Ma et al., 2024). The effects of NGF in PC12 cells were also potentiated by puerarin by activating the ERK1/2 and PI3K/AKt pathway (Gopathy et al., 2024). The natural flavonoid, astilbin has been found to reduce depressive behavior by activating MAPK/ERK and PI3K/AKt pathways in mouse models (Gopathy et al., 2024).

#### 3.3.3 Activation of Nrf2 signaling pathway

**FIGURE 6:**
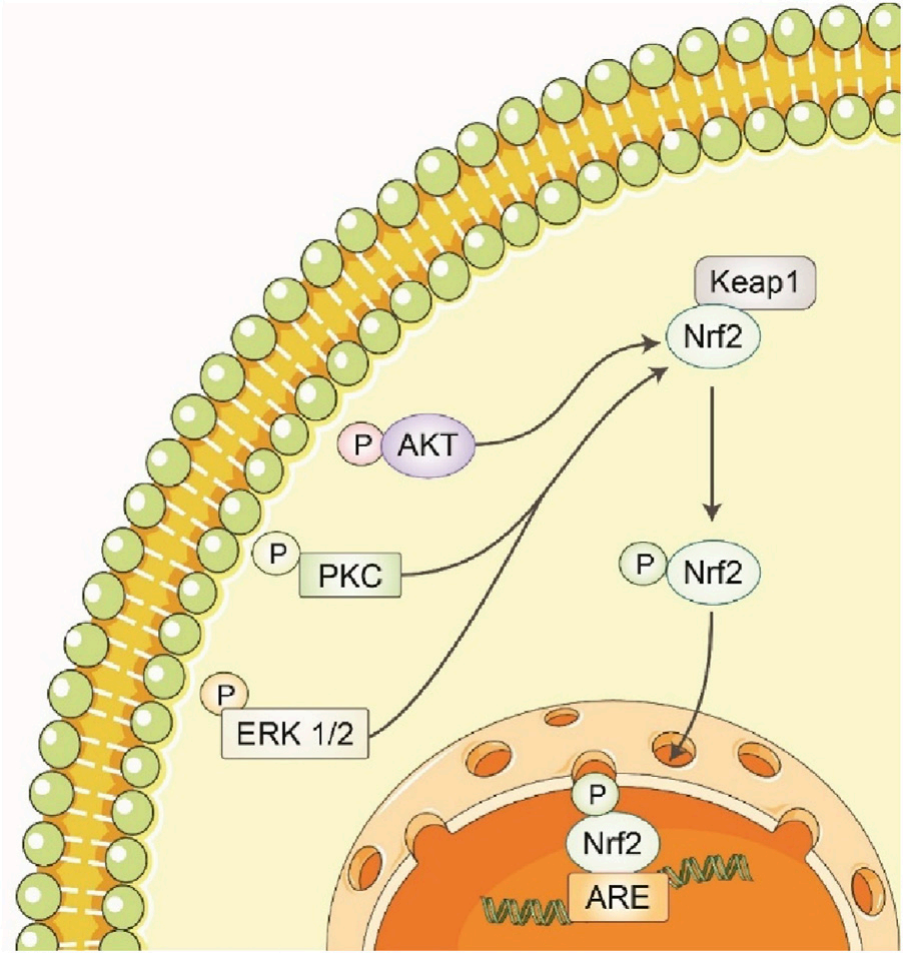
Polyphenols increase the expression of detoxification/antioxidant enzymes by activating the Keap1/Nrf2/ARE pathway. Polyphenols stimulate detoxification and antioxidant enzymes via the Keap1/Nrf2/ARE pathway. Nrf2 moves to the nucleus when Keap1 changes conformation after polyphenol contact. Nrf2 increases detoxification and antioxidant defense enzyme expression by binding to the antioxidant response element (ARE) in target gene promoters. This mechanism helps polyphenols protect cells from oxidative damage and promote health.

Polyphenols stimulate the activation of nuclear factor erythroid 2-related factor 2 (Nrf2), which in turn increases the production of detoxifying enzymes and proteins that play a role in the cellular antioxidant response (Laddha et al., 2024). Neuronal cells respond to oxidative stress through the Nrt2 pathway and the enzyme including glutathione peroxidase, γ-glutamylcystine synthetase, gluta-thione s-transferase, Ho-1, NADPH quinine oxireductase-1, thlo redoxin, sulfiredoxin, peroxiredoxin and theoredoxin reductase plays a critical role in cell defense (Cadenas-Garrido et al., 2024; Anjo et al., 2024). The Nrf2 pathway and these enzymes mainly have a role in regulating CNS disease (Xiang et al., 2024). The luteolin and puerarin are phenolic compounds that enhance HO-1 expression in PC12 cells and CAPE dopaminergic neurons by promoting Nrf2 binding to ARE (Li J. et al., 2024) (Figure 6). Through the activation of the transcription factor Nrf2, the expression of HO-1 can be increased, the EGCG was capable of protecting cells against oxidative stress (Chi et al., 2024). The neuroprotective effect of puerarin on lesioned substantia nigra caused by 6-OHDA was protested neurons in the substantia nigra by modulating BDNF expression and triggering the Nrt2/ARE pathway (Li et al., 2023). The conventional sage plants and carnosic acid have been found in neuroprotective effects by activating Nrt2 and PC125 cells (Mirza et al., 2023).

### 3.4 Proteostasis and autophagy

Polyphenols, a heterogeneous collection of naturally occurring chemicals present in plants, have a substantial impact on regulating proteostasis and autophagy, which are essential mechanisms in neuroinflammation and neurodegenerative disorders (Rahman et al., 2023). Protein homeostasis is the equilibrium of protein levels maintained by the processes of synthesis, folding, and degradation (Lippi and Krisko, 2024). In order to improve proteostasis, polyphenols stimulate the correct folding of proteins and trigger autophagy pathways, which are cellular mechanisms responsible for breaking down and recycling damaged proteins and organelles (Rahman et al., 2022). Polyphenols facilitate the removal of misfolded proteins and aggregates by autophagy, therefore mitigating neuroinflammation and slowing down the advancement of neurodegenerative disorders (Niu et al., 2024). By regulating important signaling pathways, polyphenols have therapeutic potential in the treatment of neurodegenerative disorders such as Alzheimer’s and Parkinson’s diseases (Li L. et al., 2024).

### 3.5 Neurotrophic factors

Polyphenols have the ability to increase the production of neurotrophic factors, specifically BDNF, which promotes the development, survival, and differentiation of neurons (Carito et al., 2015). Polyphenols may modulate neurotrophic factors, which could potentially impact cognitive performance and mood control (Morton et al., 2024). For instance, elevated levels of BDNF are linked to enhanced cognitive function and decreased symptoms of depression (Kong et al., 2024). Moreover, the gut-brain axis significantly mediates the impact of polyphenols on neurotrophic factors (Liu H. et al., 2024). The gut microbiota can convert polyphenols into bioactive compounds that traverse the blood-brain barrier and affect central nervous system (CNS) functioning (Domínguez-López et al., 2024). These interactions may alleviate cognitive decline and mood problems by enhancing BDNF expression and diminishing neuroinflammation (Rahmati-Dehkordi et al., 2024). Recent evidence indicates that polyphenols enhance BDNF synthesis in conjunction with physical exercise and calorie restriction. This combinatorial method presents a viable way to postpone or avert age-related cognitive deterioration and mood disorders (Aziz et al., 2024). Consequently, polyphenols embody a natural, multifarious strategy for preserving neuronal health and augmenting neurotrophic factor function. Continued investigation of their particular molecular targets and enduring effects on cerebral health is an essential area of emphasis.

### 3.6 Epigenetic modifications

Polyphenols have the ability to affect the way genes are expressed by using epigenetic mechanisms, such as DNA methylation and histone modification (Castillo-Ordoñez et al., 2024). These mechanisms can change the expression of genes that are related to the protection and degeneration of the nervous system. The diverse effects of polyphenols on cell signaling as well as cellular processes make them highly attractive for the development of therapeutic approaches to combat neurodegenerative disorders (Islam et al., 2024). Besides, DNA methylation, polyphenols can also affect histone alterations, including acetylation and methylation. Histones are proteins that encase DNA, and their alteration can either facilitate or obstruct gene transcription (Sabi, 2024). Polyphenols such as epigallocatechin gallate (EGCG) and resveratrol have been documented to influence histone acetyltransferases and histone deacetylases (HDACs), the enzymes that regulate the acetylation status of histones (Bontempo et al., 2024). Through the regulation of these enzymes, polyphenols facilitate chromatin relaxation, hence enhancing accessibility for transcription factors to activate genes that contribute to cellular repair and neuroprotection.

Additionally, polyphenols may control non-coding RNAs, including microRNAs, which are essential for the regulation of gene expression (Wu et al., 2024). Polyphenols can affect the expression of particular microRNAs implicated in neuroinflammation, apoptosis, and autophagy, thus offering a method to regulate pathways related to neurodegeneration (Prasanth et al., 2024). Notwithstanding the encouraging outcomes from *in vitro* and animal research, clinical trials investigating the epigenetic effects of polyphenols remain scarce. Future study must concentrate on determining the ideal dosages, bioavailability, and long-term impacts of polyphenols on human health. Ultimately, comprehending the manner in which polyphenols alter the epigenome may facilitate the creation of innovative, non-invasive treatments for the prevention or postponement of neurological diseases.

## 4 Recent targeting polyphenols to protect neuroinflammation and neurodegeneration

Neuropathic inflammation, a prevalent characteristic observed in several neurodegenerative disorders like Alzheimer’s disease, Parkinson’s disease, and multiple sclerosis, is distinguished by the stimulation of glial cells, the secretion of pro-inflammatory cytokines, and consequent impairment of neuronal function (Abd‐Nikfarjam et al., 2023). Polyphenols possess anti-inflammatory and antioxidant characteristics, rendering them highly promising contenders for the amelioration of these harmful processes (Sun et al., 2024). One of the principal mechanisms via which polyphenols exert their neuroprotective effects is by modulating oxidative stress, a significant contributor to neuroinflammation (Tavan et al., 2024). Polyphenols exhibit robust antioxidant characteristics, enabling them to counteract the harmful effects of reactive oxygen species (ROS) and mitigate excessive oxidative harm to neurons (Hu et al., 2024; Yahia et al., 2024). Through the process of scavenging free radicals, these entities contribute to the preservation of neuronal cell integrity and the inhibition of pro-inflammatory signaling pathways activity. Table 3 that is provided presents a comprehensive overview of several polyphenols, including their natural origins, experimental models employed for investigating their impacts, and the molecular mechanisms through which they exert neuroprotective benefits. It is becoming more widely acknowledged that these chemicals provide promising therapeutic promise in the management of neuroinflammatory and neurodegenerative disorders.

**TABLE 3:** Polyphenol sources, experimental models, and neuroprotective actions.

Polyphenol	Sources	Experimental model	Molecular action of neuroprotection	References
Curcumin	Turmeric (Curcuma longa)	Animal models of Alzheimer’s disease	Inhibits Aβ aggregation, reduces oxidative stress, modulates microglial activity	Azzini et al. (2024)
Resveratrol	Grapes, red wine	Rodent models of Parkinson’s disease	Activates SIRT1, enhances mitochondrial function, reduces inflammation	Tao et al. (2024)
Epigallocatechin gallate (EGCG)	Green tea (Camellia sinensis)	Animal models of Huntington’s disease	Inhibits apoptosis, reduces oxidative stress, and inflammation	Li et al. (2024c)
Quercetin	Apples, onions, berries	Animal models of ischemic stroke	Reduces inflammation, inhibits excitotoxicity, and oxidative stress	Shi et al. (2024)
Luteolin	Celery, parsley, thyme	*In vitro* and *in vivo* models of neuroinflammation	Suppresses microglial activation, reduces pro-inflammatory cytokines	Madhubala et al. (2024)
Kaempferol	Green leafy vegetables, tea	Rodent models of traumatic brain injury	Antioxidant activity, inhibition of NF-κB pathway	Silva dos Santos et al. (2021)
Apigenin	Chamomile, parsley	Animal models of Alzheimer’s disease	Modulates GABAergic activity, reduces amyloid plaques, anti-inflammatory effects	Charrière et al. (2024)
Anthocyanins	Berries (blueberries, raspberries)	Animal models of cognitive impairment	Inhibits oxidative stress, reduces neuroinflammation, enhances synaptic plasticity	Tran and Tran (2021)
Hesperidin	Citrus fruits (oranges, lemons)	Rodent models of aging	Increases antioxidant defenses, inhibits neuroinflammation, enhances cognition	Pontifex et al. (2021)
Baicalein	Scutellaria baicalensis (Baikal skullcap)	Animal models of Parkinson’s disease	Inhibits α-synuclein aggregation, reduces oxidative stress, anti-inflammatory	Melrose (2023)
Fisetin	Strawberries, apples, onions	Rodent models of aging and neurodegeneration	Antioxidant activity, reduces inflammation, modulates pathways involved in aging	Rauf et al. (2023)
Genistein	Soybeans, legumes	Animal models of neurodegeneration	Reduces oxidative stress, modulates estrogen receptors, inhibits neuroinflammation	Dogra et al. (2023)
Naringenin	Grapefruit, citrus fruits	Animal models of Alzheimer’s disease	Inhibits Aβ aggregation, reduces oxidative stress, and inflammation	Choi et al. (2023)
Pterostilbene	Blueberries, grapes	Rodent models of cognitive decline	Enhances antioxidant defenses, modulates sirtuins, reduces neuroinflammation	Qu et al. (2023)
Catechin	Green tea, cocoa	Rodent models of neuroinflammation	Reduces oxidative stress, inhibits pro-inflammatory cytokines, protects neurons	Özduran et al. (2023)

Curcumin, derived from turmeric (*Curcuma longa*), provides neuroprotection by inhibiting amyloid-β (Aβ) aggregation, reducing oxidative stress, and modulating microglial activity (Azzini et al., 2024). Resveratrol, found in grapes and red wine, activates SIRT1, enhances mitochondrial function, and reduces inflammation. Epigallocatechin gallate (EGCG), from green tea (*Camellia sinensis*), inhibits apoptosis while also reducing oxidative stress and inflammation (Tao et al., 2024). Quercetin, sourced from apples, onions, and berries, reduces inflammation, inhibits excitotoxicity, and decreases oxidative stress (Shi et al., 2024). Luteolin, present in celery, parsley, and thyme, suppresses microglial activation and reduces pro-inflammatory cytokines (Madhubala et al., 2024). Kaempferol, available in green leafy vegetables and tea, exhibits antioxidant activity and inhibits the NF-κB pathway (Silva dos Santos et al., 2021). Apigenin, from chamomile and parsley, modulates GABAergic activity, reduces amyloid plaques, and has anti-inflammatory effects (Charrière et al., 2024). Anthocyanins, found in berries like blueberries and raspberries, inhibit oxidative stress, reduce neuroinflammation, and enhance synaptic plasticity (Tran and Tran, 2021). Hesperidin, abundant in citrus fruits such as oranges and lemons, increases antioxidant defenses, inhibits neuroinflammation, and improves cognition (Pontifex et al., 2021). Baicalein, derived from *Scutellaria baicalensis* (Baikal skullcap), inhibits α-synuclein aggregation, reduces oxidative stress, and has anti-inflammatory properties (Melrose, 2023). Fisetin, found in strawberries, apples, and onions, exhibits antioxidant activity, reduces inflammation, and modulates pathways involved in aging (Rauf et al., 2023). Genistein, sourced from soybeans and legumes, reduces oxidative stress, modulates estrogen receptors, and inhibits neuroinflammation (Dogra et al., 2023). Naringenin, present in grapefruit and citrus fruits, inhibits Aβ aggregation, reduces oxidative stress, and inflammation (Choi et al., 2023). Pterostilbene, found in blueberries and grapes, enhances antioxidant defenses, modulates sirtuins, and reduces neuroinflammation (Qu et al., 2023). Catechin, sourced from green tea and cocoa, reduces oxidative stress, inhibits pro-inflammatory cytokines, and protects neurons (Özduran et al., 2023). These polyphenols contribute to neuroprotection by acting as antioxidants, reducing inflammation, and modulating pathways implicated in neurodegenerative diseases.

## 5 Future perspectives of polyphenols in neuroinflammation and neurodegenerative treatment

It is recommended that future investigations prioritize the elucidation of the precise molecular targets associated with polyphenols, the enhancement of their bioavailability, and the comprehension of their interactions with the gut-brain signaling pathway (Gong et al., 2024). Furthermore, the advancement of treatments based on polyphenols, potentially in conjunction with nanotechnology for precise dosage administration, has the potential to significantly transform the management of neuroinflammatory disorders (Fang et al., 2024). Furthermore, it is imperative to conduct clinical research in order to ascertain the effectiveness and safety of polyphenols in human populations, particularly when considering their long-term usage (Bolat et al., 2024). The potential incorporation of polyphenols into dietary guidelines or as dietary supplements may emerge as a pivotal approach in mitigating or decelerating the advancement of neuroinflammatory disorders, hence fostering enhanced aging outcomes and an elevated standard of living.

Recent research indicates that polyphenols have the ability to penetrate the blood-brain barrier, which is an essential characteristic for any substance that protects the brain (Isabel et al., 2024). Studies have demonstrated that they can regulate important signaling pathways linked to neurodegeneration, such as diminishing oxidative stress and inflammation, which play a crucial role in the advancement of neurodegenerative diseases (Nájera-Maldonado et al., 2024). In addition, polyphenols have the ability to prevent the clumping together of amyloid-beta peptides, which is a characteristic feature of Alzheimer’s disease (Gujarathi et al., 2024). They also improve autophagy, which helps in the removal of damaged proteins and organelles. Subsequent investigations are expected to prioritize the examination of the bioavailability and delivery mechanisms of polyphenols, given that their inadequate absorption and quick metabolism presently restrict their medicinal efficacy (Abbasi et al., 2024). Nanotechnology-based delivery methods and synthetic analogs that enhance bioavailability show great potential (Lv et al., 2024). Furthermore, comprehending the synergistic impacts of mixtures of polyphenols and their interaction with other elements in the diet could improve their effectiveness (Zhang W. et al., 2024). Additionally, clinical trials are necessary to determine the safety, effectiveness, and most effective doses of polyphenols in people (Chen et al., 2024c). As our knowledge of the molecular pathways that cause neurodegeneration increases, polyphenols may become essential elements of treatment efforts that target several factors. Therefore, the potential of polyphenols in the treatment of neurodegenerative diseases shows promise for the future. Further investigation is necessary to completely understand and utilize their benefits in clinical applications.

## 6 Potential limitations of polyphenols in neuroinflammation and neurodegeneration

Polyphenols, while showing potential in the treatment of neuroinflammation, encounter limitations such as low bioavailability, instability in the gastrointestinal tract, and fast metabolism, which minimize their therapeutic effectiveness. Moreover, the presence of inconsistent absorption and distribution patterns across individuals, the possibility of toxicity at high dosages, and the scarcity of clinical data on the long-term effects present obstacles for the application of these substances in neuroinflammatory disorders. Although they possess encouraging neuroprotective effects, their clinical application is hindered by various obstacles. A major obstacle that needs to be addressed is the issue of bioavailability. Polyphenols frequently exhibit limited absorption rates and undergo rapid metabolism within the human body, hence constraining their capacity to reach and exert their effects on specific regions within the brain (Grabska-Kobyłecka et al., 2023). The blood-brain barrier, a selective membrane, exacerbates this problem by limiting the passage of certain therapeutic substances, such as polyphenols, into the central nervous system (Carecho et al., 2024). Another issue to consider is the variety in polyphenol concentration among various plant sources, which might result in inconsistencies in dosage and effectiveness (Wang C.-W. et al., 2024). The efficacy of polyphenols can be influenced by variables such as the composition of the diet, the process of digestion, and variations in individual metabolism, which complicates the establishment of uniform treatment (Martemucci et al., 2024). In addition, although polyphenols possess antioxidant and anti-inflammatory characteristics, the intricate nature of neurodegenerative disorders, which encompass multiple pathological factors, may necessitate more focused and precise therapies beyond the capabilities of polyphenols alone (Islam et al., 2024). Further research is required to examine the interplay between polyphenols and other medications, as well as their possible adverse effects, especially in the context of prolonged usage. Translating findings from *in vitro* and animal models to clinical practice has been difficult due to the lack of replication of identified effects in human studies. To overcome these constraints, it is essential to enhance the delivery systems, conduct thorough clinical trials, and get a deeper understanding of the interactions of polyphenols. These efforts are critical in exploring the potential of polyphenols in the treatment of neurodegenerative diseases.

Future research should concentrate on enhancing the bioavailability and stability of polyphenols to overcome their limitations in neuroinflammation and neurodegeneration. A possible strategy involves the creation of sophisticated delivery methods, such as nanoencapsulation or liposomal formulations, which can improve the absorption of polyphenols and enable their passage across the blood-brain barrier. By safeguarding polyphenols from fast metabolism and degradation, these systems can enhance the concentration of active chemicals in the brain, hence augmenting their therapeutic efficacy.

Another research option is identifying particular polyphenols that have enhanced bioavailability and more targeted effects on neuroinflammatory pathways. Integrating polyphenols with additional substances or therapies may enhance their effectiveness, providing a more holistic approach to treating neurodegenerative disorders. Furthermore, it is imperative to examine the pharmacokinetics and pharmacodynamics of polyphenols in clinical studies to enhance comprehension of their interactions with other drugs and long-term safety profiles.

Standardizing polyphenol dosages according to clinical evidence from rigorously conducted human trials may mitigate variations in their efficacy. Moreover, emphasizing personalized medicine strategies that account for individual genetic variations and metabolic discrepancies may result in more accurate and efficacious treatments for neurodegenerative disorders. Ultimately, surmounting these hurdles necessitates a cooperative endeavor among academics, physicians, and pharmaceutical corporations to realize the complete therapeutic potential of polyphenols for neuroinflammation and neurodegeneration.

## 7 Conclusion

Polyphenols, naturally occurring plant metabolites, are powerful antioxidants with anti-inflammatory properties. They act on several signaling pathways, including ERK, PI3K, Nrf2/HO, PPAR, HIF, and STAT, while inhibiting pro-inflammatory biomarkers. Polyphenols also reduce oxidative stress by upregulating antioxidant enzymes like catalase and superoxide dismutase and downregulating pro-apoptotic proteins, thereby supporting neuron survival. Furthermore, polyphenols suppress acetylcholinesterase, a key enzyme involved in Alzheimer’s pathology, and chelate metals, which helps manage prion diseases. These compounds are promising for their neuroprotective effects, low toxicity, and potential to prevent neurodegenerative disorders.
